# CoVar: A generalizable machine learning approach to identify the coordinated regulators driving variational gene expression

**DOI:** 10.1371/journal.pcbi.1012016

**Published:** 2024-04-17

**Authors:** Satyaki Roy, Shehzad Z. Sheikh, Terrence S. Furey

**Affiliations:** 1 Department of Genetics, University of North Carolina, Chapel Hill, North Carolina, United States of America; 2 Departments of Medicine and Genetics, Center for Gastrointestinal Biology and Disease, University of North Carolina, Chapel Hill, North Carolina, United States of America; 3 Departments of Genetics and Biology, Center for Gastrointestinal Biology and Disease, University of North Carolina, Chapel Hill, North Carolina, United States of America; Universita degli Studi di Torino, ITALY

## Abstract

Network inference is used to model transcriptional, signaling, and metabolic interactions among genes, proteins, and metabolites that identify biological pathways influencing disease pathogenesis. Advances in machine learning (ML)-based inference models exhibit the predictive capabilities of capturing latent patterns in genomic data. Such models are emerging as an alternative to the statistical models identifying causative factors driving complex diseases. We present CoVar, an ML-based framework that builds upon the properties of existing inference models, to find the central genes driving perturbed gene expression across biological states. Unlike differentially expressed genes (DEGs) that capture changes in individual gene expression across conditions, CoVar focuses on identifying *variational* genes that undergo changes in their expression network interaction profiles, providing insights into changes in the regulatory dynamics, such as in disease pathogenesis. Subsequently, it finds *core genes* from among the nearest neighbors of these variational genes, which are central to the variational activity and influence the coordinated regulatory processes underlying the observed changes in gene expression. Through the analysis of simulated as well as yeast expression data perturbed by the deletion of the mitochondrial genome, we show that CoVar captures the intrinsic variationality and modularity in the expression data, identifying key driver genes not found through existing differential analysis methodologies.

## Author summary

In tissues and cells, changes in local environmental conditions, including the onset of disease, can result in cellular reprogramming affecting the expression and regulation of many genes. Differential expression analyses of individual genes may simply identify transient expression changes but not more important alterations in regulatory patterns. Changes in coexpression relationships of groups of genes involved in a common cellular process may indicate more fundamental changes in the cellular state that resulted in increased or decreased activity in the cellular process in response to the different conditions. Connections between related genes can be inferred by patterns in gene expression profiles and modeled as gene regulation networks. Determining how interactions in these gene expression networks have been altered by a change in condition may provide additional biological insights not captured by differential expression analyses of single genes. We developed a method, CoVar, that identifies core genes within modules whose gene network relationships are the most significantly changed between two conditions. We demonstrate CoVar’s capabilities on simulated as well as yeast expression datasets from normal and mitochondria-depleted samples where the identified genes are involved in processes known to be affected by mitochondrial depletion. Importantly, we report several genes that would not have been detected by independent differential analysis yet play key roles in the response to this perturbation.

## Introduction

The advent of high-throughput genomic data acquisition techniques has generated immense interest in the statistical and data-driven analysis of biological and biochemical interactions [1]. One such approach, network inference, attempts to identify network topologies that capture the “interactome”, defined as the set of direct or indirect molecular interactions, within a biological system [2,3]. It employs a range of computational models, such as Bayesian, autoregression, and differential equations, to answer questions ranging from basic cell biology to disease pathogenesis. The efficacy of the resultant biological networks largely depends on the ability of the computational models to capture the complex dynamics among the entities determined by nonlinear and stochastic interactions [4].

Efforts to carry out network analysis of gene expression data show that the genes acting as key players in biological processes are often highly connected nodes in the corresponding networks [5,6]. Thus, a measure of the functional significance of a gene is its ability to have paths that reach many genes in the network. On the other hand, there have been attempts to group genes into modules based on similarity in expression profiles and infer the aggregate biological function of these modules [7,8]. The computational methods to identify such modules, such as the Weighted Correlation Network Analysis [9] and Sub-Network Enrichment Analysis algorithm (SNEA), reveal subnetworks with potential regulators as well as target regulated genes, often using information from known databases of genetic interactions, where multiple regulator genes can coordinate to influence several cellular processes [10]. Thus, *coordination* and *reachability* are two necessary facets of biological network analysis.

There are predominantly two classes of network inference approaches. The first is to infer the network structure characteristics from the genomic data. For instance, co-expression networks are constructed by connecting genes with highly similar or correlated gene expression profiles [11]. Liu et al. attempted to find accurate protein-protein interaction networks, by adding and removing existing edges based on topological and semantic similarity between proteins [12]. Werhli et al. presented a graphical Gaussian model that builds a conditional independence relationship among genes under the assumption that the data follow multivariate Gaussian distribution [13] and Murphy et al. proposed a directed acyclic graph-based where directed edges capture the interaction among genes [14]. Steuer et al. used mutual information on the expression profile of genes as a measure of the regulatory interaction between them [15]. The second class of network inference leverages known associations to make inferences. Christley et al. incorporated prior transcription factor-gene interaction into gene network inference [16]. Siahpirani et al. introduced a regulatory network inference algorithm that integrates expression information from auxiliary datasets [17]. Li et al. included prior biological knowledge in gene networks by adding associations between gene pairs based on gene ontology, protein-protein interaction, and gene regulatory networks [18].

Key genes responsible for altered cellular activity between biological states are often predicted by determining individual genes whose expression profiles are significantly altered, but these changes may be better analyzed at a network level to capture effects across multiple genes and pathways linked to fundamental changes in the cellular state. To identify differences between networks from two different datasets or conditions, Zhou et al. presented an approach that creates a joint inference of two gene regulatory networks (GRNs) by fusion of gene expression and genetic perturbation data to facilitate the identification of the differential GRN under two conditions [19]. Tu et al. conceived a differential network where differential edges exist only if at least one of the two involved genes is differentially expressed. They showed that the hub nodes estimated by the model have significant biological roles [20]. Nitsch et al. introduced a network analysis tool that identifies disease-causing genes under the assumption that such genes are surrounded by differentially expressed genes [21]. Network centrality measures, such as degree, betweenness, and page rank centrality, are often used to identify the most well-connected genes, which, when removed from the network, can disrupt key functional properties of the biological processes being modeled [6, 22]. Langfelder et al. utilized network clustering analysis to identify modules of interrelated genes with similar functional roles [9], while Kogelman et al. proposed a probabilistic graphical model to elucidate transcriptomic pathways and regulators genes involved in disease pathogenesis [23]. Khwaja et al. and He et al. attempted to eliminate the strong and dominant clusters identified by existing community detection algorithms to be able to identify weak or hidden clusters within networks [24,25].

In this work, we present a unified machine learning (ML)-based approach, called *CoVar*, that builds upon the key features of existing network inference models to identify the central genes that appear to be most significantly and coordinately perturbed across gene expression datasets from distinct biological conditions. An important difference is that existing inference methods rely on modules of correlated gene expression profiles or on prior associations to guide the identification of central hub genes. In contrast, CoVar leverages a set of genes, termed *variational*, that exhibit a significant relative difference in interactions with neighbor genes across the control and perturbed expression datasets. Thus, CoVar focuses on *changes in expression relationships*, rather than commonalities in expression profiles, to infer modules of coordinated factors associated with perturbation. CoVar first creates directed networks independently in each condition using only the gene expression data. It employs an ML-based network inference approach called GENIE3 [26, 27] that captures the strength of direct or indirect interactions between genes and helps identify the *variational genes*. Unlike differentially expressed genes (DEGs), in which we may simply observe a change in expression across the control and perturbed conditions, the variational genes vary in expression with respect to their neighbors representing a more central change in the cellular state. CoVar creates a *nearest neighbor network* that includes the variational genes and their strongly connected neighbor genes. Within this network, distinct subnetworks or modules that display a high degree of intra-module connectivity and a low or no degree of inter-module connectivity are then determined. Finally, a subset of genes, termed *core genes*, are identified within each module that exhibits both coordination through strong mutual interaction and reachability with short paths to nearest neighbor network genes within the module. Such modules suggest an alteration in the interaction profile and the regulation of key biological processes being altered in the perturbed state with their core genes playing an essential role in facilitating their change in activity. We demonstrate the features and the capabilities of CoVar in identifying this inherent modularity and variationality using both simulated expression data as well as data from experiments on yeast focusing on the effects of mitochondrial genome depletion.

## Methods

### 2.1 RNA-seq data preprocessing

**Fig 1:**
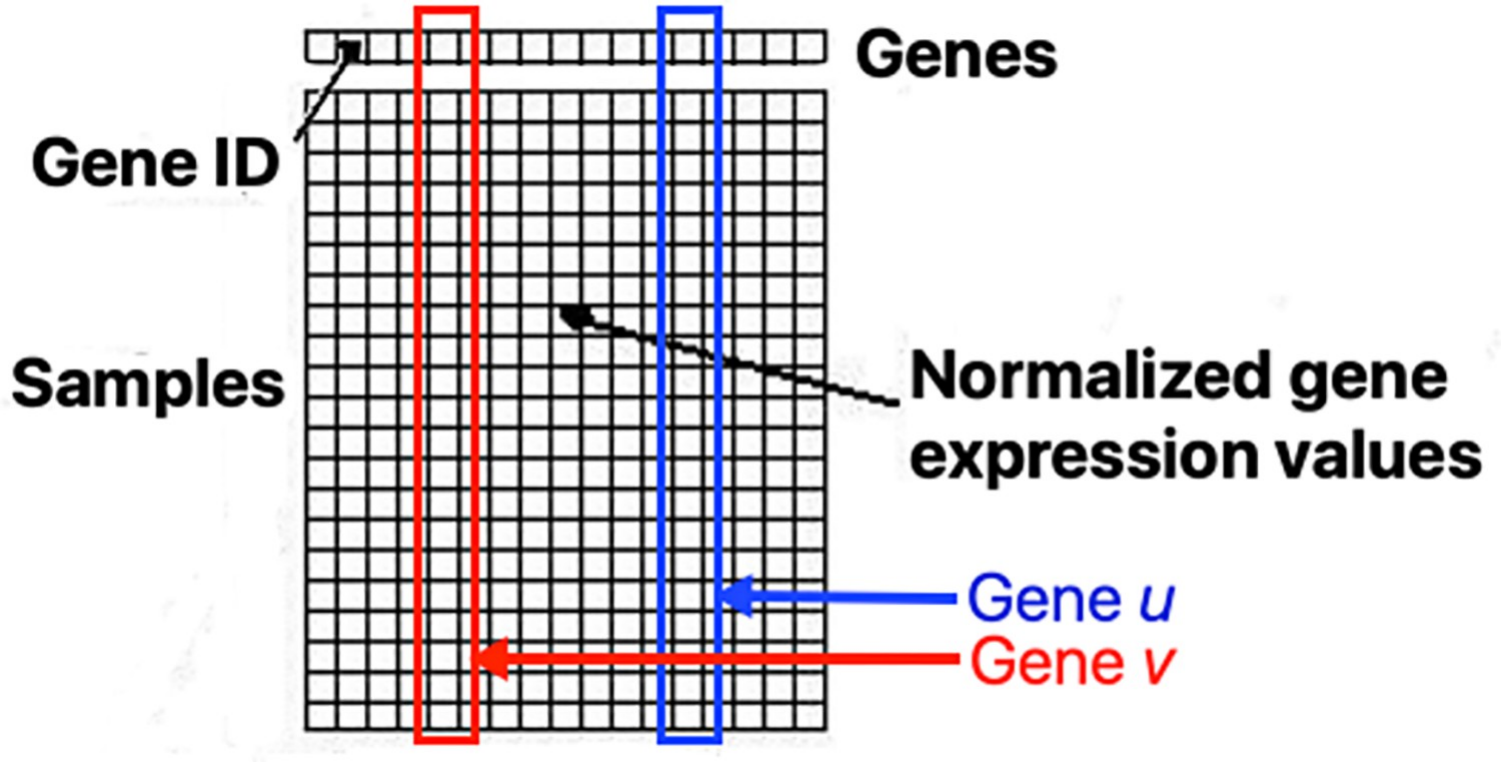
Count matrix ***X*** with shortlisted genes as columns (red and blue column represent genes ***u*** and ***v***), samples as rows, and normalized gene expression as values.

CoVar takes as input results from two sets of genome-wide expression experiments, where one set of experiments represent a perturbation in relation to the second set of control experiments. Data from each experiment is represented as *S*×*N* expression matrix ***X*** with *S* samples and *N* genes, where rows are samples and columns are gene names, i.e., ***X***_*i*,*u*_ the expression value of gene *u* in sample *i* (see Fig 1).

As a preprocessing step, genes *u* that do not satisfy a prespecified cut-off of mean expression and M-value across all samples ${[X}_{0,u},X_{1,u},\ldots ,X_{S-1,u}]$ are eliminated. The M-value is a measure of variability in gene expression based on the premise that the expression ratio of two stably expressed genes should be similar in all samples [28]. In other words, given expression matrix ***X*** and two stable genes *u*_1_, *u*_2_ in samples *j*_1_, *j*_2_, will follow $\frac{X_{j_{1},u_{1}.}}{X_{j_{1},u_{2}.}}\approx \frac{X_{j_{2},u_{1}.}}{X_{j_{2},u_{2}.}}$. Based on this, the M-value is defined in the following two steps. First, the pairwise variation between gene *u*_1_ and gene *u*_2_ is calculated as the standard deviation of the log-fold changes between their expression levels across all samples:

$$\Delta_{u_{1},u_{2}}=\sigma \left(\log\frac{X_{0,u_{1}.}}{X_{0,u_{2}.}},\log\frac{X_{1,u_{1}.}}{X_{1,u_{2}.}},\log\frac{X_{2,u_{1}.}}{X_{2,u_{2}.}},\ldots ,\log\frac{X_{S-1,u_{1}.}}{X_{S-1,u_{2}.}}\right)$$

Second, the M-value of gene *u*_1_ is calculated as:

$$M_{u_{1}}=\frac{\sum_{i1}\Delta_{u_{1},u_{i}}}{N-1}$$

This filtering is performed to better focus on genes that are at least minimally expressed, indicating they have a non-trivial role in the system, while also showing that they are affected at least in part by the perturbation.

### 2.2. *Creation of perturbed and control networks through GENIE3*

Given RNA-seq gene expression data from two biological conditions, CoVar leverages GENIE3 [26] feature selection to create networks independently for each condition. Feature selection techniques, such as logistic regression and extra-trees classification, select a subset of dimensions (or *features*) that contribute towards the prediction of an observed variable (called the *label*). These approaches often eliminate features with a low variation as they are non-informative with respect to the labels. The selection of a subset of informative features makes these techniques different from dimension reduction approaches (e.g., principal component analysis) or compression (e.g., information theory-based methods) that alter the original representation of the variables [29,30].

The choice of GENIE3 as an inference tool was guided by two reasons. Unlike regression or Bayesian methods, decision tree-based machine learning techniques like GENIE3 take fewer parameters. Moreover, the *feature ranking* used by GENIE3 helps identify a subset of genes whose expression pattern affects a given gene, thereby lending itself to the nearest-neighbor network generation module (discussed in Methods 2.4) of CoVar.

**Fig 2:**
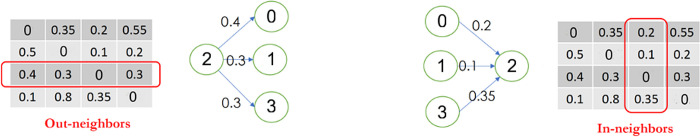
Out- and in-neighbor of gene 2 identified based on weights learnt from GENIE3.

For any (control or perturbed) gene expression data (***X***), CoVar calculates the weighted directed genie matrices (Fig 2) for each network, where each directed edge *(u*, *v)* has the weight *w*_(*u*,*v*)_∈[0,1], denoting the strength of the influence on the expression of node *v* by *u*. GENIE3 learns these weights, based on the assumption that the expression profile of gene *u* (*x*_*u*_) is a weighted function *f* of the expression of all genes *v* (*u*≠*v*) plus random noise *ϵ*, i.e.,

$$x_{u}=f(X_{-u})+\epsilon$$

Here ***X***_−***u***_ is the vector containing the measurement of all vectors except *x*_*u*_, i.e., $\left[x_{0},x_{1},\ldots ,x_{u-1},x_{u+1},\ldots ,x_{N-1}\right]$.

GENIE3 employs machine learning feature ranking to find the parameters to function *f* (which are also inter-gene influence weights *w*), by minimizing the error ${\left(x_{u}-f(X_{-u})\right)}^{2}$. The inferred influence is represented as a directed network with *N* genes as nodes and directed weighted edges from each node to all other nodes except itself.

### 2.3. Identification of variational and differentially expressed genes

CoVar leverages the weights (*w*_*u*,*v*_ signifying the extent of influence of node *u* on *v*) in the GENIE network to identify variational genes whose neighborhoods in perturbed and control networks vary the most. For each gene *u*, CoVar creates an out-degree GENIE3 weight vector $\left[w_{\left(u,0\right)},w_{\left(u,1\right)},\ldots ,w_{\left(u,N-1\right)}\right]$ and in-degree weight vector $\left[w_{\left(0,u\right)},w_{\left(1,u\right)},\ldots ,w_{\left(N-1,u\right)}\right]$, respectively. Variational genes are defined as those exhibiting the highest variation in the in- and out-neighbor weights between the control and perturbed datasets. To gauge variationality, CoVar creates concatenated (in- and out-neighbor) weight vectors, separately for both control and perturbed denoted by *V*^*control*^(*u*) and *V*^*perturb*^(*u*), respectively.

$$V^{control}(u)=[w_{\left(u,0\right)}^{control},w_{\left(u,1\right)}^{control},\ldots ,w_{\left(u,N-1\right)}^{control},w_{\left(0,u\right)}^{control},w_{\left(1,u\right)}^{control},\ldots ,w_{\left(N-1,u\right)}^{control}]$$

$$V^{perturb}(u)=[w_{\left(u,0\right)}^{perturb},w_{\left(u,1\right)}^{perturb},\ldots ,w_{\left(u,N-1\right)}^{perturb},w_{\left(0,u\right)}^{perturb},w_{\left(1,u\right)}^{perturb},\ldots ,w_{\left(N-1,u\right)}^{perturb}]$$

The variationality of gene *u* is the *mean squared error* (MSE) of *V*^*control*^(*u*) and *V*^*perturb*^(*u*), i. e., *MSE* (*V*^*control*^(*u*), *V*^*perturb*^(*u*))). For example, Fig 2 shows in red the in- and out-neighborhoods of gene 2, in the control network. The concatenated in- and out-neighbor vector for gene 2 will be *V*^*control*^(2) = [0.2,0.1,0,0.35,0.4,0.3,0,0.3]. Similarly, CoVar will create a *V*^*perturb*^(2) and estimate variationality as *MSE* (*V*^*control*^(2), *V*^*perturb*^(2))). Overall, variationality measures the amount of change in edge weights of any given gene and its neighbors in GENIE networks across different conditions. As discussed below (Methods 2.9), we contrast the variational genes with differentially expressed genes determined independently of changes in the expression of other genes. A differentially expressed gene (DEG) is one whose observed change in read count or expression value across two experimental conditions is statistically significant. For our analysis, we use the *DESeq2* [31] differential expression package and consider genes with an adjusted p-value <0.05 to be differential.

### 2.4. Constructing the nearest neighbor network

Nearest neighbor (NN) is a machine learning classification technique in which a data point is assigned a class based on the class membership of the majority of its most similar (or nearest) data points [32]. CoVar employs NN to identify genes with the strongest influences on or are strongly influenced by the variational genes and constructs the nearest neighbor network constituting the variational genes, their neighbors, and their shared directed edges.

CoVar assigns equal importance to all the variational genes. This approach greedily selects the neighbors with the highest incoming and outgoing edge weights across all the variational genes. It initializes the NN network with variational genes of the perturbed network, iteratively adds the nodes with the highest incoming or outgoing edge weight to the existing NN network and stops when there are edges in the NN network. While this greedy approach is used to carry out the experiments, we present another NN construction approach (refer to Section A in S1 Text). Note that, unlike the GENIE3 perturbed and control networks, the NN network is not fully connected. It possesses edges with the highest weights (*w*) among the variational genes and their neighbors.

### 2.5. Identification of modules through community detection

Community detection methods are designed to locate communities of similar nodes based on network structure [33, 34]. CoVar identifies modules in the nearest neighbor network using the Girvan-Newman algorithm that finds communities by progressively eliminating edges from a network [35]. At each step, the algorithm first determines betweenness across edges, measured as the importance of an edge between genes in terms of the number of shortest paths between other gene pairs the edge intercepts. It then removes the edge with the highest betweenness and recalculates the betweenness of the remaining edges. This process is repeated until the required number of modules emerge. CoVar ignores the edge directions while finding network modules.

### 2.6. Core network formation

The *k-core* of an undirected graph is the subgraph remaining where all nodes with degree less than *k* are removed. All the nodes in the *k*-core have a degree at least equal to *k* and the *k*+1-th core is always a subgraph of the *k*-th core [36]. Core genes identified by CoVar possess two properties, namely *coordination* and *reachability*. Coordination is measured in terms of graph density, defined as the ratio between the number of edges and the maximum number of edges in a directed core graph *G* (*V*, *E*). It is calculated as:

$$D_{G}=\frac{\left|E\right|}{\left|V|\times (|V|-1\right)}$$

The reachability of a directed core network *G* (*V*, *E*), *R*_*G*_, is the fraction of total nodes in the overall network that have a path from at least one core node in *u*∈*V*. We employ the *undirected* core detection, where each core gene *u* has degree *deg*(*u*) = *k*, i.e., connections with *k* other core nodes. There is also a *directed* core variant of CoVar (refer to Fig A in S1 Text), which preserves the directionality of the genes in the nearest neighbor network.

### 2.7. Combining variational, nearest neighbors, modules, and core genes across multiple runs

There may be slight variation in the variational and core genes in independent runs of the CoVar approach. This is because GENIE operates on randomized trees and the directed network it returns may vary slightly in edge weights across runs. We address this variability by combining the nearest neighbor networks across multiple runs to generate an integrated nearest neighbor network, by applying the following three-step process:

1. The integrated network is initialized as an empty network and populated with all the nodes and edges appearing in all the runs.
2. The edges that appear in less than a prespecified integral parameter *ρ* (where 0≤*ρ*≤ number of runs) are removed from the integrated network. The isolated nodes, i.e., nodes that are not connected to any other nodes in the trimmed (integrated) network are removed from it.
3. The variational, nearest neighbor, and core genes in the combined network are determined as described above (2.3–2.6).

For all experiments performed in this study, we used the parameter settings summarized in Table 1.

**Table 1:** Parameter settings used in CoVar evaluations.

Parameter	Value
M-value cut-off	0.32
Mean expression cut-off	60
Number of perturbed and control samples	3, 3
Number of trees in feature selection	10000
Percentile cut-off for variational genes	97.5
Percentile cut-off for nearest neighbor edges (*Z*)	99.95
Number of runs	25
Nearest neighbor approach	Greedy
Core network approach	Undirected

Notably, the mean expression and M-value parameters were determined by plotting the number of resulting genes based on different values of these parameters (see Fig B in S1 Text). We found that the number of genes passing these filters was most affected by the M-value, and that the gene count was significantly reduced in a small range (0.25–0.5) of M-values. We chose 0.32 as the final M-value cutoff to focus on approximately the top 50% of genes most affected by the perturbation.

### 2.8. Small world networks

Small world networks are networks in which most nodes are not neighbors of one another, and any node is reachable from any other node in a few hops (edges) [37]. The Barabási Albert preferential attachment growth model is a small world network generation approach in which we start with a few densely connected nodes, and subsequently, nodes are iteratively introduced into the network *G* (*V*, *E*), where *V* is a finite, non-empty set of objects called vertices (or nodes); and *E* is a (possibly empty) set of 2-vertex subsets of *V*, called edges [38]. Any new node *v* prefers to be attached to a well-connected node *u* in *G* with probability given by:

$$P_{G}(e(u,v))=\frac{\deg^{+}\left(u\right)+k}{\sum_{x}\deg^{+}\left(x\right)+k}$$

**Fig 3:**
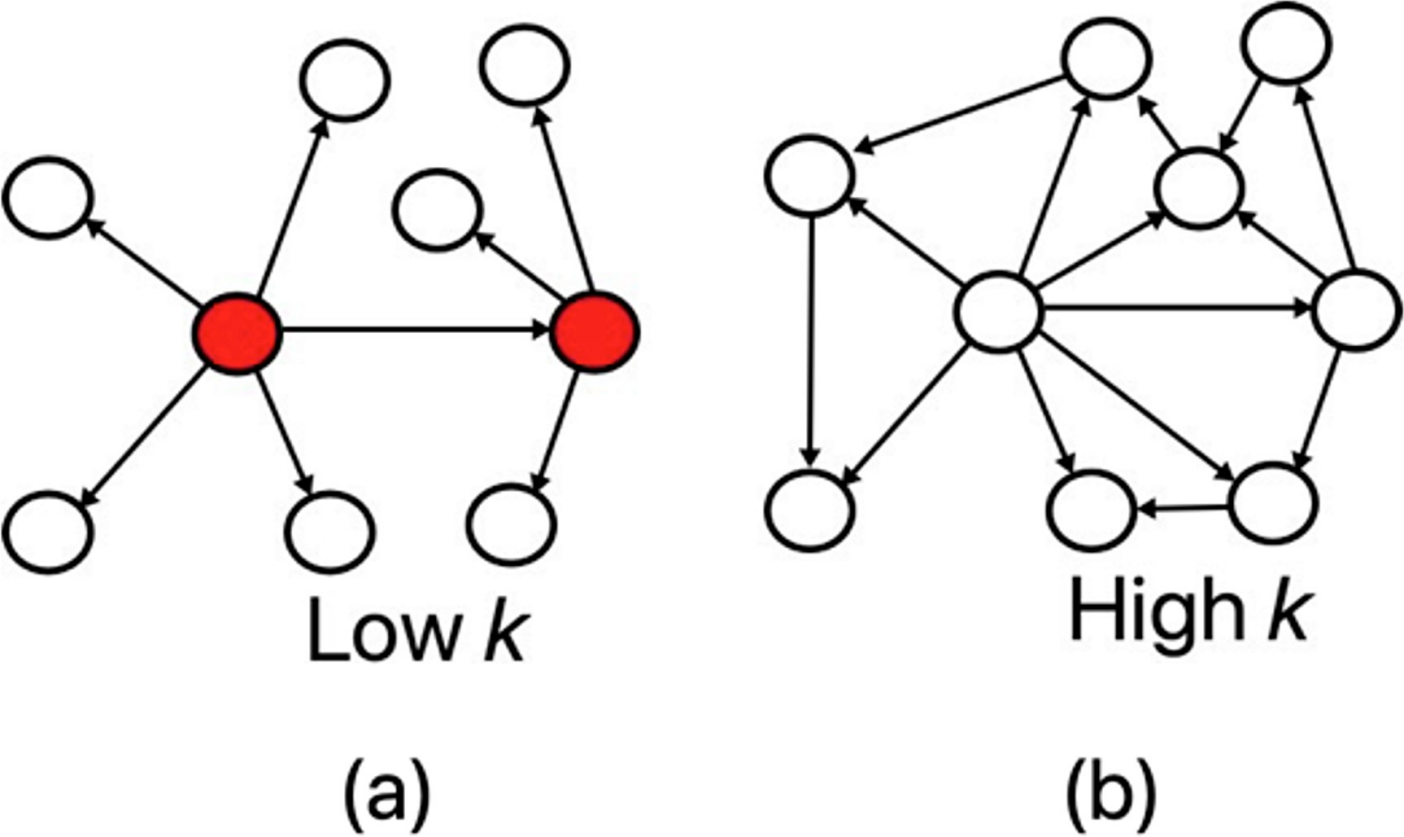
Small world networks. Networks with (a) a low value of the small world parameter *k* results in few highly connected nodes (shown in red); and (b) a high value of *k* creates networks with most nodes having similar connectivity.

Here deg^+^(*u*) (and deg^−^(*u*)) denote the out-degree (and in-degree) of node *u* and *k* is the small world parameter that assumes integral values and controls the overall connectivity of *G* (*V*, *E*). Specifically, a low value of *k* results in networks where very few nodes are highly connected (shown in red in Fig 3A); conversely, higher *k* generates networks where most nodes have average connectivity (Fig 3B).

### 2.9. Comparing differential expression with variationality

The identification of key genes driving a perturbed molecular state is most often done by determining genes that are significantly *differentially expressed*, focusing on each gene independently without considering whether or how a gene may affect or be affected by other genes. On the other hand, we define a gene as *variational* if the mean squared error of its neighborhoods in the GENIE perturbed networks varies with respect to its control network (see Methods 2.3). These suggest genes where changes in the regulatory landscape is altering expression relationships across related genes.

**Fig 4:**
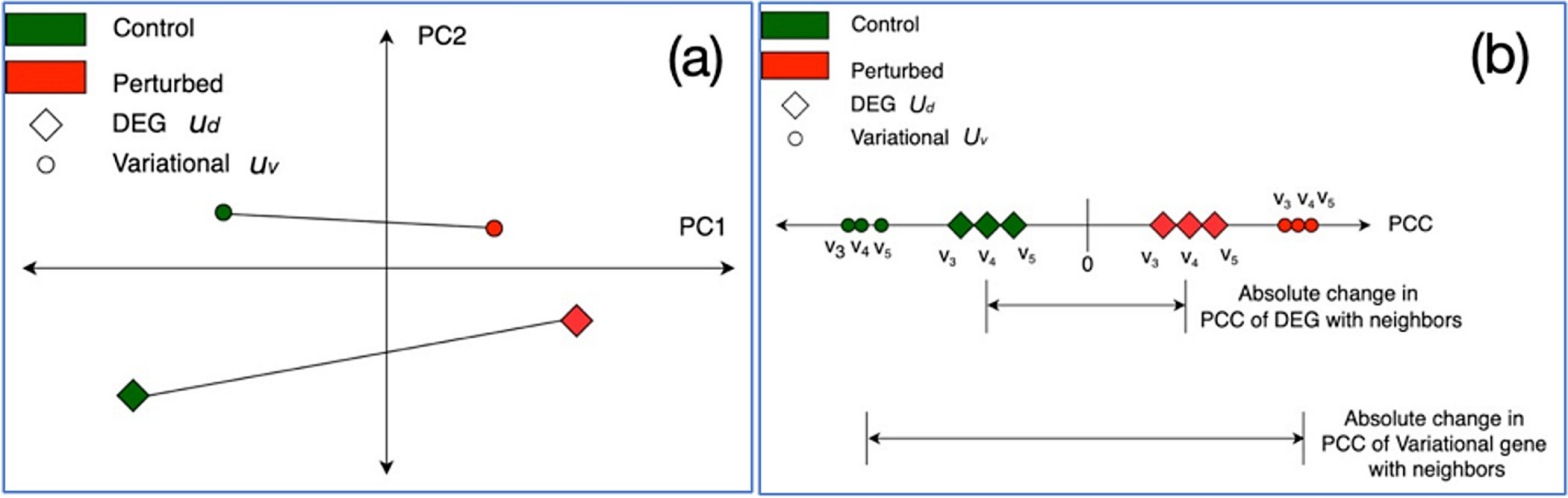
Proposed model of differences between variational and differentially expressed genes in control versus perturbed expression data. (a) Principal component analysis of expression vectors of DEG and variational gene *u*_*d*_ and *u*_*v*_, where we expect differentially expressed genes individually show greater change across conditions than variational genes; (b) Mean change in correlation between expression of *u*_*d*_, *u*_*v*_ and the neighbor genes, where we expect that variational genes will exhibit a greater change in this correlation.

Consider a DEG and a variational gene, *u*_*d*_ and *u*_*v*_, with expression vectors denoted as $x_{u_{d}}$ and $x_{u_{v}}$. A principal component analysis (PCA) using these vectors would focus primarily on the change in expression of the single gene. Fig 4A shows the first two components (PC1 and PC2) from a hypothetical principal components analysis on $x_{u_{d}}$ and $x_{u_{v}}$ in the control and perturbed expression data samples, we denote by $PC_{x_{u_{d}}}^{control},\;PC_{x_{u_{d}}}^{perturb},\;PC_{x_{u_{v}}}^{control},\;PC_{x_{u_{v}}}^{perturb}$. We intuit that there will a greater Euclidean distance between $PC_{x_{u_{d}}}^{control}$ and $PC_{x_{u_{d}}}^{perturb}$ for the DEG than between $PC_{x_{u_{v}}}^{control}$ and $PC_{x_{u_{v}}}^{perturb}$ for the CoVar variational gene. This is because the DEGs undergo a change in their *own* expression profiles in the control dataset with respect to the perturbed dataset. These expression changes captured by differential expression alone do not necessarily indicate that the regulation of those genes or how they affect the expression of other genes has changed between the two states and may represent a transient effect rather than a more fundamental cellular change.

On the other hand, the change in the expression of variational genes with respect to their neighbors may be more indicative of a change in cellular state. This relative change of variational genes is measured by the mean absolute change in the *Pearson correlation coefficient* (PCC) in the expression of *u*_*v*_ and its neighbor genes between the control and perturbed samples. We illustrate this with an example of three other neighbor genes *v*_3_, *v*_4_, *v*_5_ in Fig 4B. We can quantify this relative change in the neighborhood of a gene across the two datasets as $S=\frac{1}{3}\times \sum_{j=3,4,5}\left|PCC_{control}(u_{v},u_{j})-PCC_{perturbed}(u_{v},u_{j})\right|$. Thus, measure variationality as a function of the relative change in expression between a given gene and other genes, caused by the combination of differential expression of the gene, its neighbors, or both. In this hypothetical example, *u*_*v*_ has greater variationality than *u*_*d*_ even though it is not as significantly differentially expressed. It is worth noting that this validation approach based on the difference in correlation is *deterministic* in nature and not subject to variability across runs. This is because the difference in correlation has been directly calculated on the expression data, serving as a measure of the change in gene expression profiles, relative to one’s neighbor genes, across control and perturbed datasets.

### 2.10. Differential coexpression analysis

Differentially coexpressed genes (DCEGs) are groups of genes whose coexpression varies across conditions. DCEGs are coexpressed in the control and not in the perturbed expression datasets, or vice versa. CoXpress [39] is a differential coexpression analysis package, which applies hierarchical clustering to find coexpressed genes in one experiment and then uses a resampling technique to determine whether the same group of genes is coexpressed in experiments. Differential gene coexpression analysis (DGCA) is another differential coexpression library. Unlike CoXpress, it finds differentially coexpresed gene pairs between two experimental conditions through Fisher’s transformation followed by post-hoc statistical analysis [40]. We also employ PageRank centrality as a measure of the importance of a gene in a differential coexpression network. PageRank centrality quantifies the importance of nodes by assigning higher centrality scores to nodes with more incoming links from other nodes, indicating their prominence in the network [41].

### 2.11. Generation of simulated gene expression data

Using a previously developed method, we consider a model produces expression data of *N* genes, where $\frac{N}{2}$ genes are attributed to one of *K* functional modules, and the remaining $\frac{N}{2}$ genes constitute the null module [42]. Considering *I* datasets each containing *N* genes and *S* samples, the expression data for each gene within a module is derived through a weighted sum of latent factors associated with both their dataset and module IDs. On the other hand, the null genes exhibit expression values determined by the weighted sum of latent factors related to their null identity and module IDs. Each latent factor adheres to a normal distribution with a mean of 0 and different standard deviations. As pointed out earlier, given that the module (or null) IDs are inherent to the generated expression data, they function as ground truth during the evaluation of CoVar in comparison to other baseline approaches.

### 2.12. Yeast validation dataset

**Fig 5:**
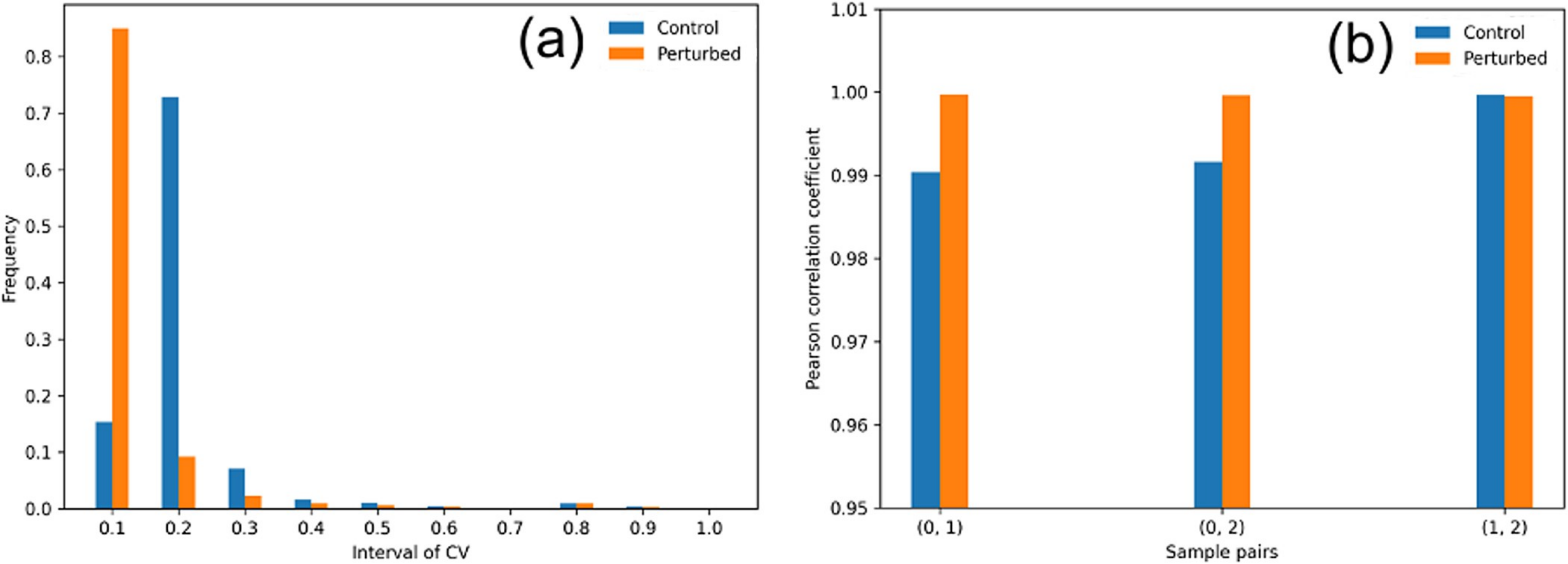
Variability in yeast expression dataset: (a) coefficient of variation; (b) pairwise Pearson correlation coefficient within the control and perturbed samples.

We validate CoVar using gene expression data from a study on mitochondrial genome depletion in yeast (GEO accession: https://www.ncbi.nlm.nih.gov/geo/query/acc.cgi?acc=GSE162197) [43]. We consider control and perturbed datasets of a wild-type and a mutant strain in which the mitochondrial genome has been depleted. The RPKM normalized values provided by this study are directly input to CoVar, without any other preprocessing measures. The *control samples* are EV1 mRNA-Seq WT replicates 1–3) GSM4946300, GSM494630, GSM4946302), while the *perturbed samples* are EV1 mRNA-Seq r0 replicates 1–3 (GSM4946315, GSM4946316, GSM4946317). Fig 5A shows that the coefficient of variation (CV), measured the ratio between standard deviation and mean, within control and perturbed samples center around 0.2 and 0.1, respectively, suggesting that there is little within-sample variability, while Fig 5B depicts that the within-sample pairwise Pearson correlation is greater than 0.99.

## Results

### 3.1. Overview of CoVar

**Fig 6:**
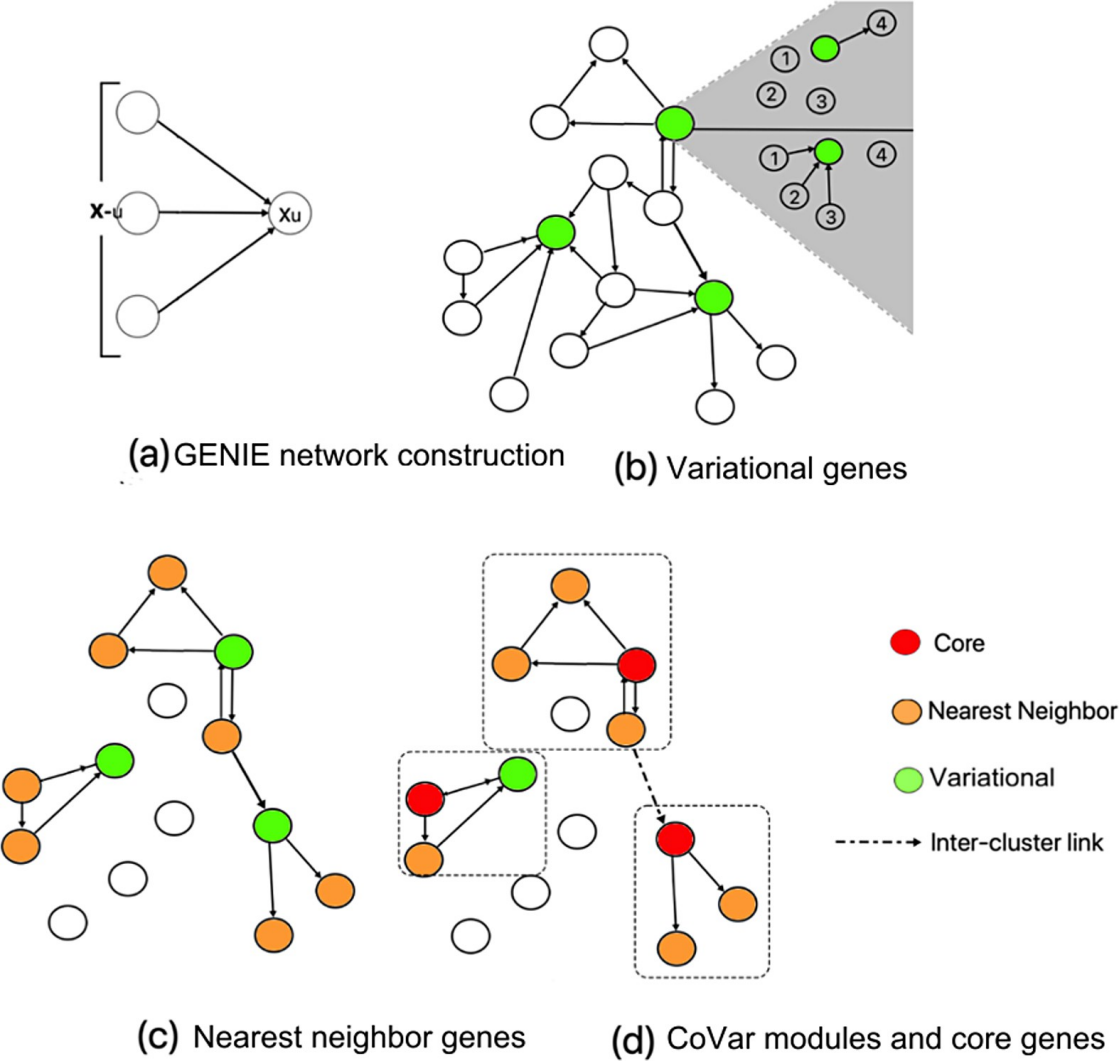
Steps in the CoVar method. (a) GENIE uses randomized trees defines the edges and weights connecting genes in the two separate conditions; (b) variational genes (colored green) are identified based on differences in edges across the control and perturbed networks; (c) nearest neighbors (colored orange) for the variational genes are identified in the perturbed network; (d) modules are defined and core genes are identified for the nearest neighbor networks contained in each module.

CoVar utilizes machine learning (ML) and network science to identify variational and core (or central) genes from gene expression data that are altered across biological conditions. Expression data from perturbed and control conditions are each represented as networks, where nodes are genes and expression relationships are directed edges (*u*, *v*) with weight *w*_*u*,*v*_∈[0,1] that represents the strength of the influence of gene *u* on the expression of gene *v* (Fig 6A; Methods 2.2). Genes that show the largest changes in the strength of influence relationships (weights) with neighbor genes are identified as variational genes (Fig 6B; Methods 2.3) providing an initial set of genes highly affected by the biological perturbation. Genes with strong connections to the variational genes, denoted as their nearest neighbors (Fig 6C; Methods 2.4), are determined, and together with the variational genes form the nearest neighbor network. Modules, or network communities, are identified to focus on well-connected groups of genes (Fig 5D; Methods 2.5). Within any module, core genes are determined that have two specific properties: (1) *coordination*, meaning they form a densely interconnected core network with other core genes; and (2) *reachability*, meaning they have directed paths to the bulk of the non-core genes, influencing the latter’s expression (Methods 2.6). As the initial perturbed and control networks are estimated using a non-deterministic ML approach, this process undergoes multiple iterations to find a set of genes consistently as identified variational, nearest neighbor, and core genes (Methods 2.7).

### 3.2. Characterization of Core Genes

We aim to identify core genes that are both highly coordinated amongst themselves while also having high reachability to other genes within the network. To determine how well we capture these characteristics, we created 1000 small world networks (see Methods 2.8) with parameters *k* = 3 and *N* = 100,200,⋯,500 nodes each and determined *c* core nodes in each network. We also randomly chose *c* core nodes and summarized the number of times that the coordination and reachability (Methods 2.6–2.7) of the randomly chosen nodes equals or exceeds those of the CoVar core nodes in the corresponding nearest neighbor network. Table 2 shows that the CoVar core nodes offer the best mix of coordination and reachability–while no randomly chosen node set has higher coordination than the core nodes, only a fraction (0.121−0.415) of these sampled sets equal (but do not exceed) the reachability of the CoVar core nodes.

**Table 2:** Number of times the coordination and reachability of randomly selected nodes in 1000 small world networks of *k* = 3 equals or exceeds those of the code nodes.

No. of nodes (*N*)	Coordination (>, ≥)	Reachability (>, ≥)
100	(0, 0)	(0, 121)
200	(0, 0)	(0, 145)
300	(0, 0)	(0, 329)
400	(0, 0)	(0, 395)
500	(0, 0)	(0, 415)

**Fig 7:**
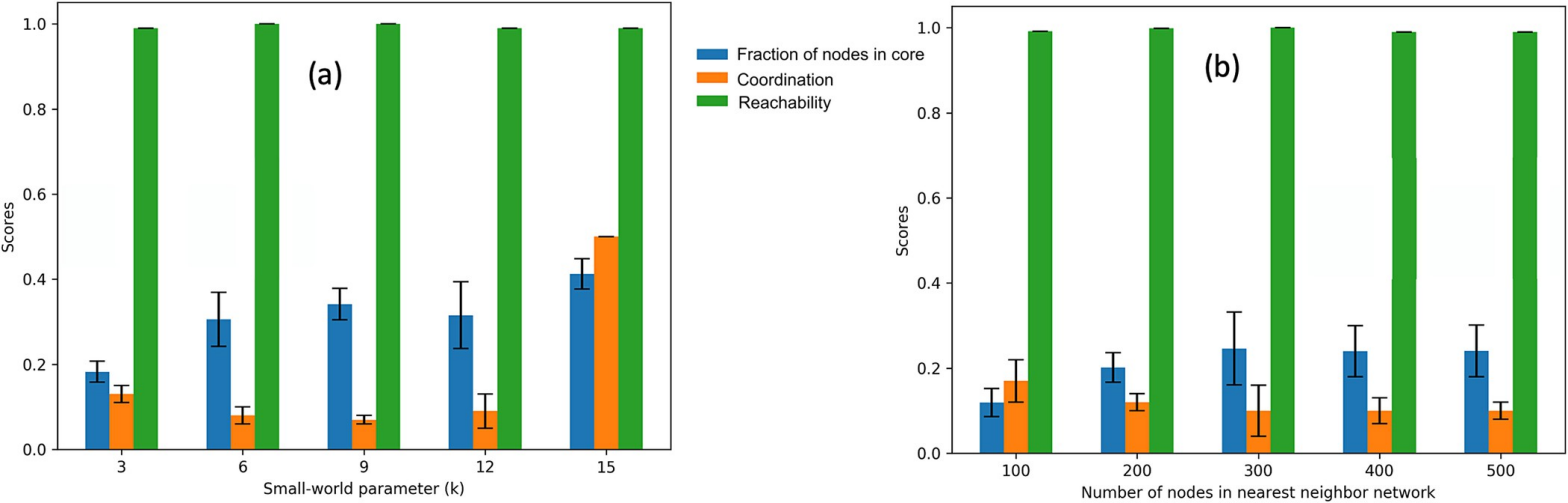
Coordination and reachability of small world networks for varied (a) small world parameter (*k*) and number of nodes *N* = 200 and (b) number of nodes and *k* = 3.

We were interested in how the distribution of the degree of node edges affects core gene identification. As described in Methods 2.8, the small world property of networks can be controlled using the parameter *k*. A small *k* generates a network with a few highly interconnected hub nodes that are connected to the majority of the non-hub nodes. Increasing *k* results in a network of uniform connectivity and fewer hubs. We found that in all cases, the reachability of the core nodes remained very high, but that the fraction of nodes of identified core nodes increased as *k* increased (Fig 7A). Interestingly, the coordination was similar across all runs except for the one with the highest *k*, where coordination was substantially increased. Furthermore, we aimed to determine whether the number of nodes in the nearest neighbor network had a significant effect on core gene identification. We varied the number of nodes *N* while keeping *k* consistent. Again, the reachability remained high across all settings for *N*, while the fraction of nodes identified as core nodes was inversely correlated with the coordination (Fig 7B). As *N* increased, the fraction of nodes in the core also increased while the coordination decreased. This shows that these basic properties of coordination and reachability of the networks are stable across network sizes.

## 3.3 Comparison against baseline approaches using simulated data

We assessed three approaches—CoXpress [39], DGCA [40], and CoVar—based on their efficacy in two crucial aspects: (a) *module identification*, demanding that the clusters identified by an approach align with the module IDs inherent in the expression data, and (b) *differential coexpression*, requiring the approach to find variations in the expression profiles of a gene group between the control and perturbed datasets. While both CoXpress and DGCA measure differential coexpression, their operational methodologies differ from each other (as well as CoVar). *CoXpress* initially employs clustering to identify modules in both control and perturbed datasets. Subsequently, within each dataset, it utilizes a statistic to compare the pairwise correlation coefficients of genes in a module to a random set of genes. If the difference (i.e., observed statistic—random statistic) is statistically significant in one dataset but not in the other, the module is considered differentially coexpressed. In contrast, *DGCA* employs Fisher’s approach for each gene pair, determining the difference in coexpression between the datasets. Once again, if this difference is significant based on a prespecified threshold, the pair is deemed differentially co-expressed.

**Fig 8:**
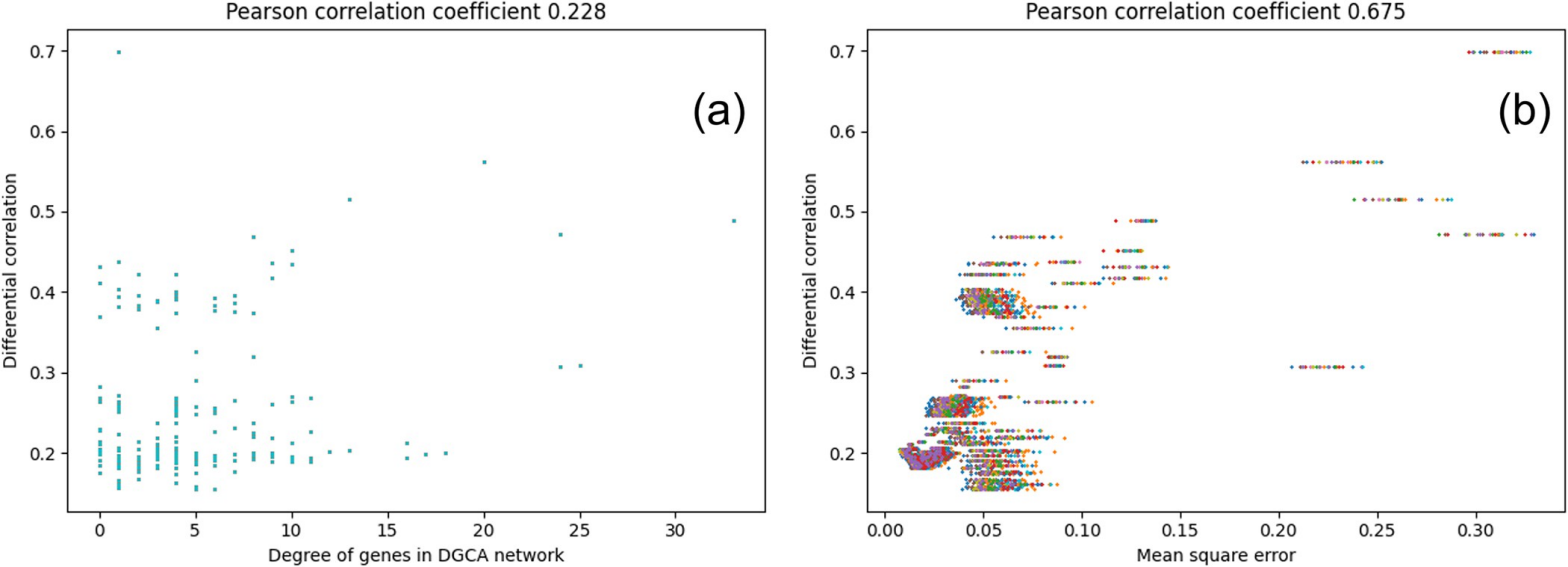
Identification of differential coexpression: (a) differential correlation of genes against their degree in the DGCA network; and (b) differential correlation of genes against their variationality (measured in terms of means square error of their weight vectors in GENIE3 networks).

We applied both DGCA and CoXpress to the simulated data of *N* = 200 genes (comprising 100 null and 100 module genes belonging to 4 modules) with 15 samples each for control and perturbed data (see Methods 2.11). We constructed a differential coexpression network based on the first 500 differentially coexpressed gene pairs. (Refer to Table A in S1 Table for the list of differentially coexpressed gene pairs for adjusted p-value cutoffs of 0.005 and 0.01 and the defined network.) As DGCA first finds differentially co-expressed gene pairs, it does not discover the inherent community structure of the expression data (as evidenced by the DGCA network diagram in Table A in S1 Table where the clusters are not well-demarcated). We assessed the variability of the key genes identified by DGCA using the same measure as detailed in Methods 2.9, the *difference in correlation*, which measures the relative change in the expression of a given gene with respect to its nearest neighbors across the control and perturbed networks. Specifically, in Fig 8, we plotted the difference in correlation of these genes against their degrees in the DGCA network, showing that DGCA exhibits a moderate capability in capturing variability in terms of Pearson Correlation Coefficient (PCC) 0.228 (see Fig 8A).

Next, CoXpress was applied to the same data. CoXpress focuses on module finding as the first step. Recall that the simulated expression data has 25 genes in each of the 4 modules and another 100 genes as part of the null model. Due to its emphasis on module finding CoXpress correctly groups many of the genes into their respective modules, but also detects modules that do not correspond to the simulated modules. CoXpress then defines differential coexpression scores of the modules, which are summarized in Table B in S1 Table, where the reported scores can be interpreted as the relative difference in coexpression of a module across the datasets, on a scale of 0(no difference) to 1 (maximum difference). As can be seen, CoXpress does not detect these modules as being highly differentially coexpressed. This may be because the coexpression relationships between genes in the modules do not change, but rather the whole module together is differentially expressed across the two conditions.

**Fig 9:**
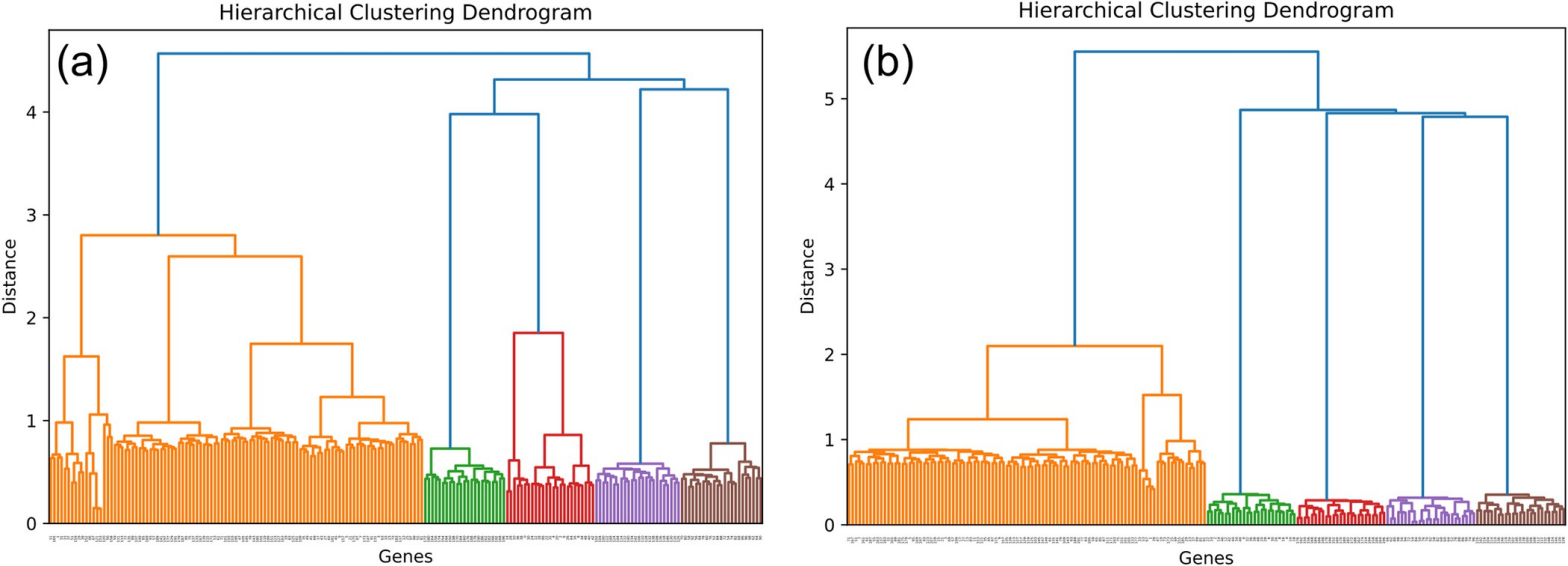
Agglomerative hierarchical clustering on the GENIE3 weights of the two simulated datasets identifies the genes belonging to the null model (colored orange) and the genes belonging to the 4 functional modules as per the simulated expression data.

Both of these approaches emphasize exactly one of the criteria we define as important in determining differentially expressed networks. Unlike DGCA and CoXpress, CoVar aims to balance both goals: *module identification* and *differential coexpression*. As highlighted in Results 3.1, CoVar systematically uncovers modules in a nearest-neighbor network centered on a limited set of variational genes. Subsequently, it identifies core genes that distinctly represent the differential coexpression patterns within these modules. This approach enables CoVar to effectively capture both modular and variational patterns in the expression data. We applied CoVar to the simulated data as detailed above (see Methods, Results 3.1), with results shown in Table C in S1 Table. As described earlier (Methods 2.2), CoVar employs GENIE3 to measure weights denoting the influence of each gene on another. When applied to the two simulated networks, there is a distinct separation of both null genes (colored orange) and modular genes belonging to the 4 modules (Fig 9A and 9B). Due to this, we find that CoVar identifies 5 modules, where three of the modules consist exactly of the 25 genes generated for modules in the simulated data, and the remaining two, consisting of 19 and 6 genes, are linked by two edges that together constitute the remaining generated module. We also investigated the correlation difference of a gene against CoVar’s measure of variationality, measured in terms of mean squared error. The resultant correlation across the 25 runs of CoVar is 0.67, which outperforms the correlation difference exhibited by the well-connected (or high degree) DGCA genes (see Fig 8B).

### 3.4. Analysis of gene networks altered by mitochondrial genome depletion in yeast

To demonstrate the ability of CoVar to identify genes that contribute to altered expression in a perturbed state, we analyzed expression data from yeast generated under two conditions: a normal state and a perturbed state in which the mitochondrial genome was deleted in a study by Lui et al. [43]. They discuss that mitochondrial dysfunction and oxidative phosphorylation (OXPHOS) impairment due to mitochondrial genome depletion leads to adaptive responses, where the downregulated processes are associated with ribosome biogenesis and the upregulated processes are mitochondrial translation, mitochondrial gene expression, and carbohydrate metabolic process. Upon deletion of the mitochondrial genome, a primary consequence is a reduction in mitochondrial membrane potential.

We filtered non-coding genes and lowly expressed genes (mean expression < 60 or M-value < 0.32) resulting in 2318 genes remaining. We carried out 25 independent runs of CoVar on 3 control and 3 perturbed samples, each of which generates 2318-node control and perturbed networks with slightly varying edge weights. For each run, the top 47 variational genes (97.5 percentile highest mean squared error (MSE)) were selected. We note that 22 out of the 25 most consistently identified variational genes across the 25 runs are also differentially expressed (p-adjusted cut-off 0.05). In each run, the nearest neighbors were then determined, and the nearest neighbor network was constructed with 547 genes on average. Within each nearest neighbor network, modules of highly connected genes were determined, with there being 3–4 modules consistently found across the 25 runs. Finally, core genes were identified within each module with an average of 63 core genes per run. Table 3 shows summary statistics of the core, nearest neighbor, and variational genes across these 25 runs, where the mean density and reachability of the core nodes in the perturbed network are 0.067 and 0.247 respectively. It is noteworthy that the mean MSE of the variational genes is nearly double that of the entire set of 2318 genes.

**Table 3:** Summary statistics of the core and variational genes across 25 runs.

Metric	Mean	St. Dev.
Density of core genes	0.420	0.117
Reachability of core genes	0.999	0.003
(Mean) MSE of variational genes	0.021	0.001
(Mean) MSE of nearest neighbor network	0.012	0.001
Mean (and standard deviation of) the number of nearest neighbor genes across 25 runs	547.52	23.73
Mean (and standard deviation of) the number of core genes across 25 runs	96.88	12.44
Mean (and standard deviation of) the number of modules across 25 runs	3.96	0.52

We compiled the data across the 25 independent runs to generate a final network of three distinct modules consisting of 267, 113, and 94 genes for a total of 474 genes (Methods 2.7; Table D in S1 Table–the table of genes). Of these, 86 genes were designated as core genes, with 45 in module 1 (M1), 24 in module 2 (M2), and 17 in module 3 (M3). We examined connections in the final network for the core genes and found that on average, each core gene had 16.99 out edges and 17.76 in edges compared to 2.75 out edges and 2.58 in edges for non-core genes. This is expected based on the focus on reachability in defining core genes.

To determine whether the genes identified across the whole network corresponded to previously published results, we performed an enrichment analysis of the set of all nearest neighbor genes using Enrichr [44]. We found that among the top gene ontology terms are translational elongation, structural integrity of the ribosome, translation termination as well as post-transcriptional modification regulated by the untranslated region of the mRNA, and protein metabolism. Indeed, these results match well with the general cellular processes cited as being affected by mitochondrial genome depletion.

To better understand the genes contained within the modules, we performed a similar pathway enrichment analysis on genes in each of these independently. Again, we see that the top GO terms and pathways for all three modules were related to the ribosome or translation, and many of the remaining were related to mitochondrial function and metabolic processes (Table D in S1 Table). As the three modules have mutually exclusive sets of genes, we assessed whether there were distinct functions associated with each module. In the first module (M1), we find enriched gene sets related to translation very generally, along with mitochondrial organization, protein degradation, and protein transport into multiple nuclear locations including the mitochondria and endoplasmic reticulum. This module includes the enzyme Mitochondrial intermembrane space Import and Assembly protein 40 (*MIA40*) gene, a key component of the MIA pathway that imports proteins to the inner mitochondria and facilitates their correct oxidative folding [45]. Module M1 also includes 20 transposable element genes. Increased transposition activity has been associated with response to stress conditions in yeast and other organisms [46]. In the second module (M2), we see distinct RNA processing functions, especially for ribosomal RNAs, but also related to respiration that include several cytochrome c oxidase (COX) genes and isocitrate dehydrogenase 2 (*IDH2*). The third module (M3) is specifically enriched for ribosomal genes involved in ribosome assembly as well as key genes in lipid, sterol, and fatty acid metabolism such as the mitochondrial NADH-cytochrome b5 reductase *MCR1*, the farnesyl diphosphate synthase *ERG20 (FPP1)*, and the C-5 sterol desaturase *ERG3*. Thus, we find that the identified modules provide important information on relationships between genes involved in more specific cellular functions than previously noted.

### 3.5. Comparison of expression change characteristics between differentially expressed genes and variational genes

Identification of key genes driving a perturbed molecular state is most often done by determining genes that are significantly differentially expressed. These analyses focus on each gene independently without considering how a gene may affect or be affected by other genes. We aimed to understand how variational genes identified by CoVar compared with differentially expressed genes. We identified differentially expressed genes (DEGs) using DESeq2 (Methods 2.3) and ranked genes based on their computed adjusted p-value. We considered genes with adjusted p-values < 0.05 to be differentially expressed.

**Fig 10:**
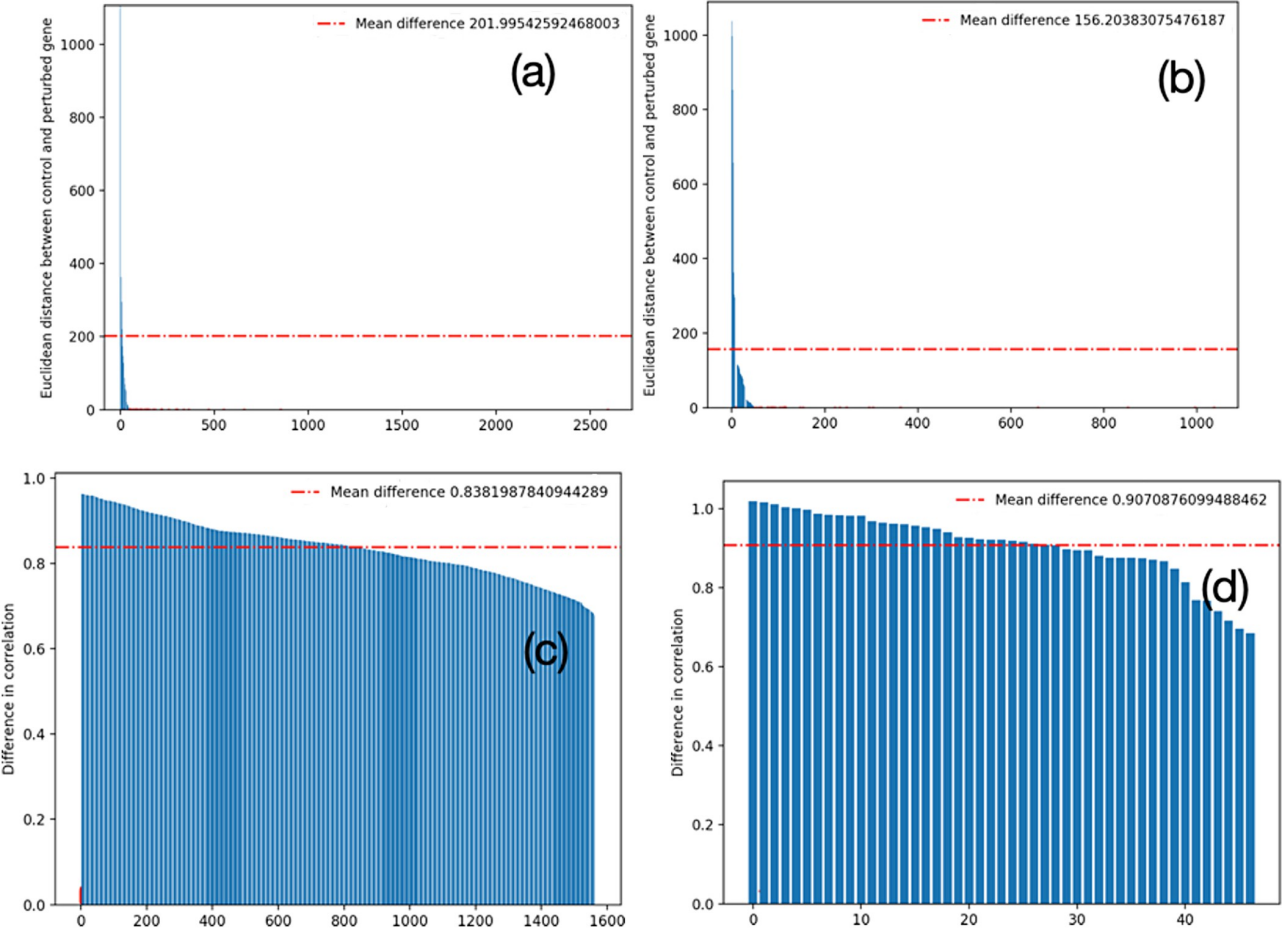
Differentially expressed genes (DEGs) versus variational genes. Principal component analysis of expression vectors of (a) DEGs (b) variational genes; Mean change in Pearson correlation between the expression of (c) DEGs and (d) variational genes against other genes.

To test this hypothesis on the yeast dataset, we carried out the above principal component analysis (PCA)- and Pearson correlation coefficient (PCC)-based study. Specifically, for any given gene, we first applied PCA and recorded the Euclidean distance between the vectors comprising the first two components (PC1 and PC2) in the control and perturbed networks. Fig 10A and 10B depict these distances for all of the DEGs and variational genes. We see that the DEGs have a higher mean distance than variational genes indicating DEGs undergo a greater change in their own expression vector between the perturbed samples and the control samples, similar to our hypothetical example. Second, we calculated the PCC for each of the DEGs and all other genes and similarly for variational genes and all other genes in one of our 25 CoVar iterations. Here, we find that the mean change in the PCC of the expression vector of the variational genes with all other genes between control and perturbed data is higher than the mean change for DEGs (0.907 vs 0.838. Fig 10C and 10D), similar to our hypothetical example. Note that the average and standard deviation in correlation change (*S*) of the variational genes across 25 runs are 0.8477 and 0.269, respectively.

**Fig 11:**
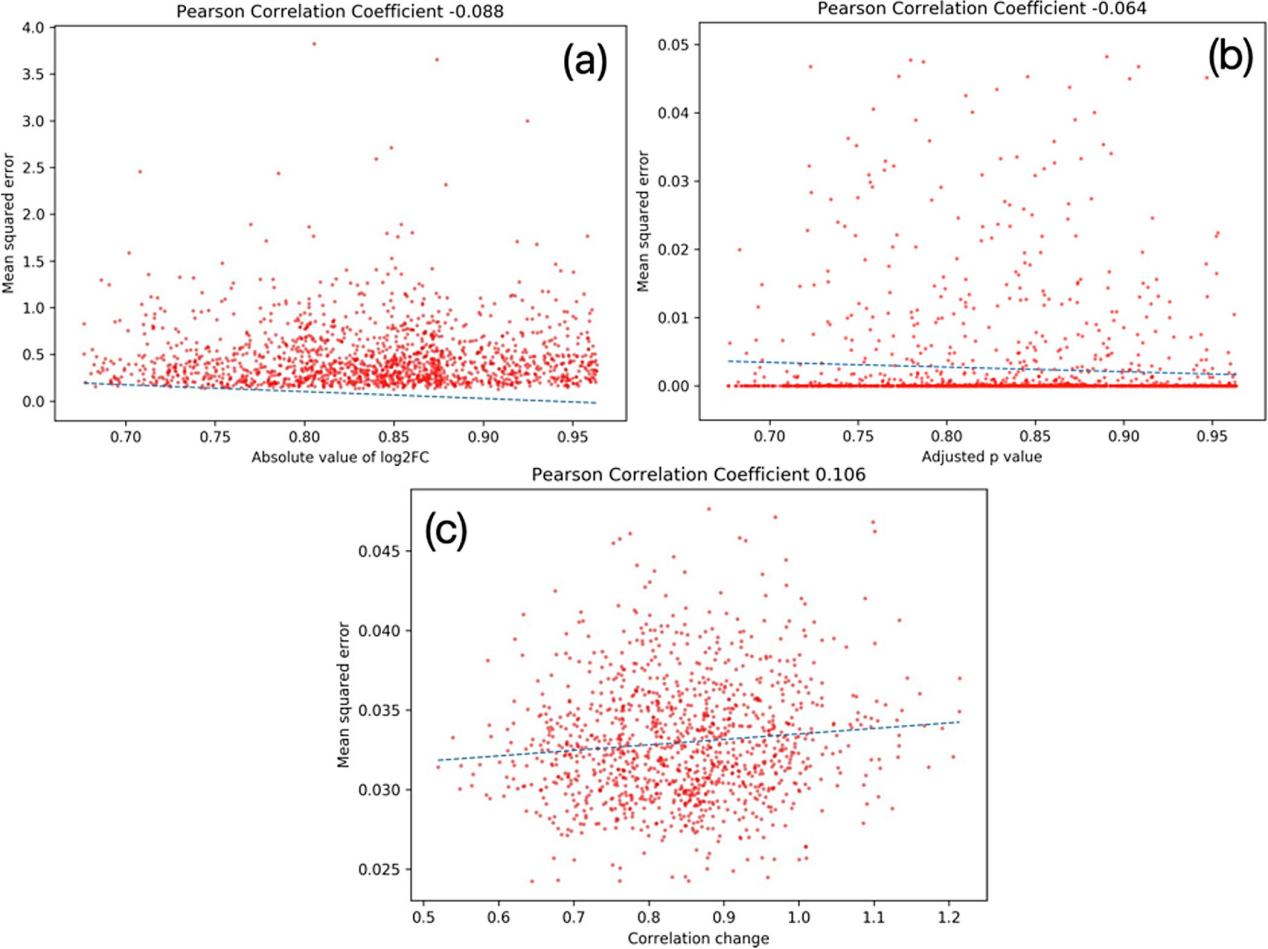
Pearson correlation between mean squared error (between the control and perturbed networks) and (a) absolute value of log fold change and (b) adjusted p value of the differentially expressed genes, as well as (c) correlation change of variational genes in the 25 runs.

We performed a direct comparison of relative change in the neighborhoods of the DEGs and variational genes in terms of the correlation change *S*. The variationality of a gene is measured in terms of the MSE of its control and perturbed weight vectors of the edges with its neighbors in the GENIE networks (Methods 2.2). The goal here is to contrast the *S* scores of the DEGs (genes likely to have a high absolute value of log_2_FC for a low adjusted value) and variational genes across the 25 runs (with higher mean MSE). Fig 11A and 11B show that the MSE of the DEGs is uncorrelated with both the absolute log_2_FC (*PCC* = -0.088) and adjusted p values (*PCC =* -0.064), while Fig 8C shows that the correlation change of the variational has a low but positive association (*PCC* 0.106) with their MSE. This supports that the variational genes exhibit a greater overall change in expression relative to the other genes than DEGs.

### 3.6 Comparison of cellular functions between differentially expressed genes and core genes

We further compared our core genes more directly with the most differentially expressed genes to see how their reported biological functions aligned with the change in experimental conditions. First, we ranked our core genes in decreasing order of their frequency of occurrence as a core gene in the 25 runs of CoVar. We also ranked the DEG genes based on their adjusted p-value to represent the significance of their differential expression. We find that 69 of the 86 core genes were differentially expressed (adjP < 0.05; Table D in S1 Table), indicating that 17 (20%) were not. Further, the average DEG ranking for core genes was 234.19. These show that while most core genes were differentially expressed, they were not simply the most differential genes.

As mentioned earlier, most of the core genes with a frequency of occurrence of at least 10 (74 out of 86) are DEGs and are amongst the most frequent variational genes. The top two ranked DEGs, Isocitrate DeHydrogenase 2 (IDH2; upregulated on mitochondrial genome loss) and Vacuolar Transporter Chaperone 3 (VTC3; downregulated), were also core genes (ranked 27^th^ and 28^th^). IDH2 is a mitochondrial enzyme that is critical for a properly functioning oxidative phosphorylation system [47]. VTC3 is one of four subunits of the vascular chain transporter that helps control polyphosphate levels, critical for energy metabolism [48]. Other than these, though, only one other top-40 ranked DEG was identified as a core gene in the final combined model. We note that other top-ranked DEGs did appear as a core gene in at least one of the CoVar iterations. For example, Heat Shock Protein 12 (HSP12), associated with heat shock, osmostress, and oxidative stress [49], ranked 3rd among DEGs and appeared in 4 runs as core genes. ARGinine requiring 3 (ARG3) is the 5th ranked DEG and a core gene in 7 runs that has been shown to be induced by heat shock and by other cellular stress inducers [50]. Finally, Multiprotein Bridging Factor 1 (MBF1), ranked 9th among DEGs and a core gene in 5 runs [51], is associated with developmental processes and abiotic stress, particularly heat stress, in plants.

Many notable core genes were not among the top-ranked DEGs or not DEGs at all. The Blocked Early in Transport 1 (BET1) gene was the most consistently chosen core gene across the 25 iterations but was only the 374th most significant DEG (Table D in S1 Table). It had the largest number of edges in the final network (21 in edges, 75 out edges). BET1 is a membrane protein involved in vesicle transport between the endoplasmic reticulum (ER) and the Golgi apparatus that is upregulated on mitochondrial genome loss. The orthologous Bet1 protein in Drosophila was determined to be necessary for the maintenance of the mitochondrial genome, with prolonged knockdown of Bet1 associated with an increase in mitochondrial membrane potential [52]. In this study, expression of BET1 is increased upon mitochondrial genome loss, presumably in an attempt to maintain a normal membrane potential. The core gene Copper TRansporter 2 (CTR2) gene (35^th^ ranked core gene), a low-affinity copper transporter that is thought to make available copper stored in vacuoles, was not differentially expressed (adjP = 0.13, Table D in S1 Table). Interestingly, the related gene Copper TRansporter 1 (CTR1), a high-affinity copper transporter and part of the final nearest neighbor network but not a core gene, was significantly upregulated. Copper is essential to several mitochondrial functions including the electron transport chain and energy metabolism. Copper deficiency has been linked to increased mitochondrial membrane potential [53]. Together, these suggest that maintenance of the membrane potential upon mitochondrial genome loss may depend on access to intracellularly stored copper and that this process is driven by CTR2, even though its relative expression level is not greatly changed.

Other notable core genes include Prohibition Complex subunit 1 (PHB1; 4^th^ ranked core gene, 176^th^ ranked DEG, upregulated), which contributes towards mitochondrial homeostasis and differentiated T cell survival [54]; Mitochondrial Ribosomal Proteins of the Large subunit MRPL20 and MRPL44 (29^th^ and 71^st^ ranked core genes, 87^th^ and 109^th^ ranked DEGs, upregulated) that are critical for protein synthesis within the mitochondrion [55]; OLEic acid requiring 1 (OLE1; 37^th^ ranked core gene, 51^st^ ranked DEG, upregulated), which is involved in mitochondria inheritance [56]; and Proteinase C1 (PRC1; 16^th^ ranked core gene, 207^th^ ranked DEG, upregulated) that regulates mitochondrial biogenesis during erythroid development [57].

### 3.7 Comparison of CoVar modules with differentially coexpressed genes

We examined the validity of the modules of covariational and neighboring genes identified by CoVar analysis by comparing them with genes identified by differential coexpression analysis using CoXpress. Specifically, we compared the overlap between the genes in the CoVar modules and genes found by CoXpress to be coexpressed in the perturbed dataset but not in the control dataset (refer to Tables E and F in S1 Table). We find that several sets of genes are both differentially coexpressed and belong to the same CoVar modules. BET1, SPC3, and VOA1 form a differential coexpression set that has a mean core frequency of 12.66 in 25 runs and appears in the same CoVar module (M1). Similarly, differentially coexpressed genes cPRC1, YNL284C-B, YPR158C-D, and AIM6 have a mean core frequency of 10.25 and appear in CoVar module M1. Also, differentially coexpressed genes DTD1 and SDH2 have a mean core frequency of 10 and are present in CoVar module M2. RPS0B, URM1, and RCR1 are differentially coexpressed, appear in CoVar module M3, and have a mean core frequency of 9.33.

We compared the number of differentially coexpressed gene pairs found by differential gene correlation analysis (DGCA) and also within similar modules in CoVar and also determined common coexpression relationships found by DGCA and CoXpress. In Table G in S1 Table, we report 2903 pairs of genes that occur as coexpressed in DGCA and appear in the same CoVar modules; similarly, in Table H in S1 Table, we report 1307 gene pairs that appear as differentially coexpressed in DGCA and CoXpress. We note, though, that both CoXpress and DGCA provide rather simple connections between two or a small number of genes, whereas CoVar produces larger modules of the order of tens to hundreds of coordinately expressed genes.

Not all of the most differentially expressed genes appeared in the final CoVar model, and we sought to better characterize these. We examined the top 50 most differentially expressed genes that were absent from nearest neighbor networks in all 25 CoVar runs. We estimated the average mean squared error (MSE) across these runs, which explains the role of these DEGs as variational genes. Of the 50, 29 were found within the initial set of 2318 genes that were required to be protein-coding genes and above a minimum expression threshold (see 3.4). The average rank of these 29 was 1293 ± 655 when sorted based on the decreasing order of MSE (Table I in S1 Table), suggesting there was little change in their coexpression patterns with other genes. Additionally, we assessed the differential coexpression ranks of these 50 genes using DGCA and CoXpress. For DGCA, we generated a network comprising differentially coexpressed gene pairs and calculated the page rank centrality of each gene as a measure of their importance within the network. We found that only 27 of the DEGs appeared in the differentially coexpressed network, and these exhibited a poor mean rank of 1200 ± 617 when sorted in the decreasing order of *PageRank centrality* (see Methods 2.10). Simultaneously, we explored whether multiple DEGs coexisted within a similar differentially coexpressed cluster identified by CoXpress. There exist only 5 and 12 genes that participate in differentially coexpressed clusters in the control dataset and the perturbed dataset with respect to a randomized network, respectively, with a mean rank of 71.8 ± 29.3 and 71.8 ± 49.2, respectively. Notably, there were no occurrences of multiple of these DEGs within the same CoXpress cluster. These observations support that while the expression of these DEGs was significantly affected by the change in condition, their regulatory neighborhoods, as captured by CoVar, remained relatively stable meaning the expression changes of these genes did not significantly alter how they interacted with other genes. Thus, despite being differentially expressed, these genes do not appear to be drivers of cellular changes at the network level.

## Discussion

We present a network analysis framework, called *CoVar*, that analyzes expression data across two different conditions to identify central genes potentially involved in more fundamental changes to the cellular state due to the perturbation. CoVar prioritizes genes whose expression seems to influence the largest number of modified coexpression relationships in a coordinated manner, not simply genes whose individual expression levels have been changed. We believe the unique strengths of CoVar will be most beneficial in instances of significant cellular reprogramming due to an altered gene regulatory landscape, such as in disease or extreme changes in environment, where reprogrammed cells will not only show changes in its expression profile but also its regulatory interactions leading to altered responses to external stimuli.

Given control and perturbed expression datasets, CoVar employs feature ranking-based machine learning to create separate networks with directed edges having weights commensurate with the influence of a gene on the expression of another. Our analysis of simulated and real expression data serves to highlight the distinctive characteristic of CoVar that enables it to capture the *modularity* and *variationality* in the expression datasets. It opts for the selectivity of a few top variational genes rather than an exhaustive list. This intentional restraint serves a dual purpose—it captures pivotal genes with significant relative differences across control and perturbed datasets while deliberately allowing space for the emergence of modularity within the network. This step facilitates the discovery of modules centered around each variational gene, ultimately leading to the identification of core genes that typify the genes influencing the underlying biological processes.

We demonstrated the efficacy of CoVar using the simulated and real expression datasets. We showed that CoVar identifies core genes that maximize coordination and reachability with other genes in the network, critical characteristics of genes with widespread influence. Our analysis of wild-type and mitochondrial genome-depleted yeast datasets further revealed that genes can be variational due to the combined effect of the differential expression of itself and its neighbor genes. Broadly, we found that the variational and core genes identified by CoVar have functions and are involved in pathways that are unsurprisingly altered upon mitochondrial genome loss, not all of which were identified in a differential expression analysis of these data. On the other hand, a significant number (20%) of the core genes do not display significant differential expression between the two conditions. Using multiple differential coexpression methods, differential coexpression analysis (DGCA) and CoXpress, we provide evidence that the differentially expressed genes not featuring in the nearest-neighbor networks of CoVar are also not differentially coexpressed relative to other genes across the control and perturbed datasets.

From a network standpoint, we showed that it is possible to quantify the coordination and reachability of core genes using network measures. *Coordination*, indicative of the density in the connectivity of the core genes, offers a measure of how tightly interconnected they are within the module, while, *reachability*, assessed by the existence of paths to non-core genes, serves as a metric for understanding the extent to which core genes can influence other genes in the module. These measures collectively contribute to a comprehensive understanding of their perceived impact on the regulatory dynamics within biological networks. This observation holds true in the context of the mitochondrial genome-depleted yeast datasets, where our analysis underscored their substantial contributions to pivotal biological pathways. The coordinated activity of these core genes implies that alterations in their expression levels may cascade to influence other genes within the module, potentially driving the observed phenotypic changes. The inputs to CoVar are simply two expression datasets across two conditions, and there are no restrictions in terms of organism or the nature of these conditions. There need not be a direct relationship between the exact samples used except that the data needs to be from the same set of genes in the same organism. Thus, CoVar can be effectively applied to expression data from diverse plants or animals, providing insights into the regulatory dynamics of different biological systems.

The limitations of CoVar are as follows. *First*, the method operates with two datasets at a time, necessitating multiple pairwise comparisons for scenarios with more than two conditions. This could amplify the analytical complexity in experiments featuring multiple conditions. *Second*, CoVar’s performance is contingent on various parameters, including cut-offs for variational, nearest neighbor, and core genes, as well as the methods for generating the nearest neighbor network and core genes. Determining optimal parameter values may pose case-specific challenges. Third, CoVar may encounter computational hurdles, especially with large-scale datasets or those involving time-series analysis that could impact the feasibility and efficiency of applying CoVar in certain scenarios.

The CoVar approach is generalizable in many ways. *First*, while our choice of GENIE3 as the network inference tool was guided by its capability of providing an exact measure of the directed regulatory influence of each gene on the other, CoVar can operate on any input network generated through other means such as co-expression, Bayesian, Gaussian, or machine learning models. While Bayesian inference models are a powerful alternative for network inference, they often present challenges such as (1) prohibitively high computational cost when applied to a large number of variables (i.e., genes) and (2) unreliability of the inferred network from a few samples [58]. A future direction is to compare GENIE3-based CoVar with alternatives that use regression, matrix factorization, and Bayesian-based inference techniques, such as variational inference like NetDiff [59] to infer gene networks.

*Second*, the application of the network centrality measures can present new insights, as we have demonstrated during our analysis. Alternatively, other measures could be used when alternative aspects of relationships between genes are deemed more important. *Third*, CoVar offers different modalities at each step. The choice of mean expression and M-value cut-off determines the size of the initial gene list and consequent variational and core genes. There is no deterministic way to define these parameters, but we do not anticipate that the primary results of an analysis would be greatly altered by slight variations in these settings. Alternatively, variational genes can be estimated based on other measures of changes between networks, for instance, based on other relationships between the in-degree and out-degree or both (Results 3.2). Nearest neighbor identification has coverage and greedy approaches, and the core network can be directed or undirected. These modes can be selected to infer varying core genes based on the number of samples as well as the number or types of entities (i.e., genes, miRNAs) of the genomic data.

*Fourth*, CoVar is extensible to adapt to time-varying datasets. In addition to analyzing the variabilities between the control and perturbed networks, it can pinpoint the variations introduced within the control and perturbed networks over time. *Finally*, there are several ways to incorporate prior knowledge about transcription, pathways, ontology, diseases/drugs, or cell types [18]. For instance, one can generate nearest-neighbor networks consisting of the known regulators of genes of interest or downstream genes of known transcription factors before the identification of the core genes. Moreover, one can find the paths in the control or perturbed networks, where the genes are connected by the highest edge weights. This approach lends itself to the analysis of other datatypes (such as chromatin, miRNA, and protein expression), where the variabilities of the combined weights of the network paths can reveal central genes specific to the perturbation.

## Supporting information

S1 Table **A.** List of DGCA gene sets in the simulated data that are also differentially coexpressed as per CoXpress. DGCA network of top 500 differential coexpressed gene pairs. **B.** List of differentially coexpressed gene sets (determined through CoXpress analysis) in the simulated data. **C.** CoVar analysis on simulated expression data. Aggregated CoVar network across 25 runs. **D.** Annotated Yeast Analysis. Variational, nearest-neighbor, and core genes identified by CoVar, along with their differential expression information. **E.** Dissimilarity between differentially expressed genes (DEGs) and CoVar genes in yeast dataset. List of highly ranked CoVar genes that are not highly differentially expressed, and vice versa. **F.** Coexpression analysis. List of differentially coexpressed gene sets (determined through CoXpress analysis) in yeast dataset that also belong to the same CoVar module. **G.** Similarity between differential gene coexpression analysis (DGCA) and CoVar analysis. List of DGCA gene sets that also belong to the same CoVar module. **H.** Similarity between differential gene coexpression analysis (DGCA) and CoXpress analysis. List of DGCA gene sets that are also differentially coexpressed as per CoXpress. **I.** Variationality in terms of mean squared error and differential coexpression the top 50 most differentially expressed genes that were absent from nearest neighbor networks in all 25 CoVar runs.
(XLSX)

S1 Text Details of nearest-neighbor and core network identification, characterization of variational and core genes, and the identification of the initial set of genes through a mean expression and M-value cut-off. (DOCX)

## References

[1] Churko J. M., Mantalas G. L., Snyder M. P., & Wu J. C. Overview of high throughput sequencing technologies to elucidate molecular pathways in cardiovascular diseases. Circulation research. 2013; 112(12), 1613–1623. doi: 10.1161/CIRCRESAHA.113.300939 23743227 PMC3831009

[2] Aalto A., Viitasaari L., Ilmonen P., Mombaerts L., & Gonçalves J. Gene regulatory network inference from sparsely sampled noisy data. Nature communications. 2020; 11(1), 3493. doi: 10.1038/s41467-020-17217-1 32661225 PMC7359369

[3] Zhang B., Tian Y., & Zhang Z. Network biology in medicine and beyond. Circulation: Cardiovascular Genetics. 2014; 7(4), 536–547. 25140061 10.1161/CIRCGENETICS.113.000123 PMC4333150

[4] Oates C. J., & Mukherjee S. Network inference and biological dynamics. The annals of applied statistics. 2012; 6(3), 1209. doi: 10.1214/11-AOAS532 23284600 PMC3533376

[5] Koschützki D., & Schreiber F. Centrality analysis methods for biological networks and their application to gene regulatory networks. Gene regulation and systems biology. 2008; 2, GRSB–S702. doi: 10.4137/grsb.s702 19787083 PMC2733090

[6] Macau E. E. Ed.; A mathematical modeling approach from nonlinear dynamics to complex systems. 2018; Springer. 22.

[7] Koschützki D., Schwöbbermeyer H., & Schreiber F. Ranking of network elements based on functional substructures. Journal of theoretical biology. 2007; 248(3), 471–479. doi: 10.1016/j.jtbi.2007.05.038 17644116

[8] De Bivort B., Huang S., & Bar-Yam Y. Empirical multiscale networks of cellular regulation. PloS computational biology. 2007; 3(10), e207. doi: 10.1371/journal.pcbi.0030207 17953478 PMC2041980

[9] Langfelder P., & Horvath S. WGCNA: an R package for weighted correlation network analysis. BMC bioinformatics. 2008; 9, 1–13. 19114008 10.1186/1471-2105-9-559 PMC2631488

[10] Saelens W., Cannoodt R., & Saeys Y. A comprehensive evaluation of module detection methods for gene expression data. Nature communications. 2018; 9(1), 1090. doi: 10.1038/s41467-018-03424-4 29545622 PMC5854612

[11] Van Dam S., Vosa U., van der Graaf A., Franke L., & de Magalhaes J. P. Gene co-expression analysis for functional classification and gene–disease predictions. Briefings in bioinformatics. 2018; 19(4), 575–592. doi: 10.1093/bib/bbw139 28077403 PMC6054162

[12] Liu J., & Qiu J. Locally Adjust Networks Based on Connectivity and Semantic Similarities for Disease Module Detection. Frontiers in genetics. 2021; 12, 726596. doi: 10.3389/fgene.2021.726596 34759955 PMC8575408

[13] Werhli A. V., Grzegorczyk M., & Husmeier D. Comparative evaluation of reverse engineering gene regulatory networks with relevance networks, graphical Gaussian models, and Bayesian networks. Bioinformatics. 2006; 22(20), 2523–2531. doi: 10.1093/bioinformatics/btl391 16844710

[14] Murphy K., & Mian S. Modelling gene expression data using dynamic Bayesian networks Vol. 104; Technical report, Computer Science Division, University of California, Berkeley, CA. 1999.

[15] Steuer R., Kurths J., Daub C. O., Weise J., & Selbig J. The mutual information: detecting and evaluating dependencies between variables. Bioinformatics. 2002; 18(suppl_2), S231–S240. doi: 10.1093/bioinformatics/18.suppl_2.s231 12386007

[16] Christley S., Nie Q., & Xie X. Incorporating existing network information into gene network inference. PloS one. 2009; 4(8), e6799. doi: 10.1371/journal.pone.0006799 19710931 PMC2729382

[17] Siahpirani A. F., & Roy S. A prior-based integrative framework for functional transcriptional regulatory network inference. Nucleic acids research. 2017; 45(4), e21–e21. doi: 10.1093/nar/gkw963 27794550 PMC5389674

[18] Li Y., & Jackson S. A. Gene network reconstruction by integration of prior biological knowledge. G3: Genes, Genomes, Genetics. 2015; 5(6), 1075–1079. doi: 10.1534/g3.115.018127 25823587 PMC4478538

[19] Zhou X., & Cai X. Inference of differential gene regulatory networks based on gene expression and genetic perturbation data. Bioinformatics. 2020; 36(1), 197–204. doi: 10.1093/bioinformatics/btz529 31263873 PMC6956787

[20] Tu J. J., Ou-Yang L., Zhu Y., Yan H., Qin H., & Zhang X. F. Differential network analysis by simultaneously considering changes in gene interactions and gene expression. Bioinformatics. 2021; 37(23), 4414–4423. doi: 10.1093/bioinformatics/btab502 34245246

[21] Nitsch D., Tranchevent L. C., Thienpont B., Thorrez L., Van Esch H., Devriendt K., et al. Network analysis of differential expression for the identification of disease-causing genes. PloS one. 2009; 4(5), e5526. doi: 10.1371/journal.pone.0005526 19436755 PMC2677677

[22] Mistry D., Wise R. P., & Dickerson J. A. DiffSLC: A graph centrality method to detect essential proteins of a protein-protein interaction network. PloS one. 2017; 12(11), e0187091. doi: 10.1371/journal.pone.0187091 29121073 PMC5679606

[23] Kogelman L. J., Cirera S., Zhernakova D. V., Fredholm M., Franke L., & Kadarmideen H. N. Identification of co-expression gene networks, regulatory genes and pathways for obesity based on adipose tissue RNA Sequencing in a porcine model. BMC medical genomics. 2014; 7, 1–16. 25270054 10.1186/1755-8794-7-57 PMC4183073

[24] Khawaja F. R., Sheng J., Wang B., & Memon Y. Uncovering hidden community structure in multi-layer networks. Applied Sciences. 2021; 11(6), 2857.

[25] He K., Soundarajan S., Cao X., Hopcroft J., & Huang M. Revealing multiple layers of hidden community structure in networks. arXiv. 2015; preprint arXiv:1501.05700.

[26] Huynh-Thu V. A., Irrthum A., Wehenkel L., & Geurts P. Inferring regulatory networks from expression data using tree-based methods. PloS one. 2010; 5(9), e12776. doi: 10.1371/journal.pone.0012776 20927193 PMC2946910

[27] Huynh-Thu V. A., & Geurts P. dynGENIE3: dynamical GENIE3 for the inference of gene networks from time series expression data. Scientific reports. 2018; 8(1), 3384. doi: 10.1038/s41598-018-21715-0 29467401 PMC5821733

[28] Vandesompele J., De Preter K., Pattyn F., Poppe B., Van Roy N., De Paepe A., et al. Accurate normalization of real-time quantitative RT-PCR data by geometric averaging of multiple internal control genes. Genome biology. 2002; 3, 1–12. doi: 10.1186/gb-2002-3-7-research0034 12184808 PMC126239

[29] Kumar V., & Minz S. Feature selection. SmartCR. 2014; 4(3), 211–229.

[30] Saeys Y., Inza I., & Larranaga P. A review of feature selection techniques in bioinformatics. Bioinformatics. 2007; 23(19), 2507–2517. doi: 10.1093/bioinformatics/btm344 17720704

[31] Love M., Anders S., & Huber W. Differential analysis of count data–the DESeq2 package. Genome Biol, 15(550). 2014; 10–1186. 10.1186/s13059-014-0550-8 PMC4302049 25516281

[32] Zhang M. L., & Zhou Z. H. A k-nearest neighbor-based algorithm for multi-label classification. In 2005 IEEE international conference on granular computing. 2005, July; (Vol. 2, pp. 718–721; IEEE.

[33] Newman M. E. Modularity and community structure in networks. Proceedings of the national academy of sciences. 2006; 103(23), 8577–8582. doi: 10.1073/pnas.0601602103 16723398 PMC1482622

[34] Dugué N., & Perez A. Directed Louvain: maximizing modularity in directed networks. 2015; (Doctoral dissertation, Université d’Orléans.

[35] Girvan M., & Newman M. E. Community structure in social and biological networks. Proceedings of the national academy of sciences. 2002; 99(12), 7821–7826. doi: 10.1073/pnas.122653799 12060727 PMC122977

[36] Hell P., & Nešetřil J. The core of a graph. Discrete Mathematics. 1992; 109(1–3), 117–126.

[37] Amaral L. A. N., Scala A., Barthelemy M., & Stanley H. E. Classes of small-world networks. Proceedings of the national academy of sciences. 2000; 97(21), 11149–11152. doi: 10.1073/pnas.200327197 11005838 PMC17168

[38] Newman M. E. The structure and function of complex networks. SIAM review. 2003; 45(2), 167–256.

[39] Watson M. CoXpress: differential co-expression in gene expression data. BMC bioinformatics. 2006; 7, 1–12. 17116249 10.1186/1471-2105-7-509 PMC1660556

[40] McKenzie A. T., Katsyv I., Song W. M., Wang M., & Zhang B. DGCA: a comprehensive R package for differential gene correlation analysis. BMC systems biology. 2016; 10, 1–25. 27846853 10.1186/s12918-016-0349-1 PMC5111277

[41] Zhang P., Wang T., & Yan J. PageRank centrality and algorithms for weighted, directed networks. Physica A: Statistical Mechanics and its Applications. 2022; 586, 126438.

[42] Wang K., Narayanan M., Zhong H., Tompa M., Schadt E. E., & Zhu J. Meta-analysis of inter-species liver co-expression networks elucidates traits associated with common human diseases. PLoS computational biology. 2009; 5(12), e1000616. doi: 10.1371/journal.pcbi.1000616 20019805 PMC2787626

[43] Liu S., Liu S., He B., Li L., Li L., Wang J., et al. OXPHOS deficiency activates global adaptation pathways to maintain mitochondrial membrane potential. EMBO reports. 2021; 22(4), e51606. doi: 10.15252/embr.202051606 33655635 PMC8025004

[44] Kuleshov M. V., Jones M. R., Rouillard A. D., Fernandez N. F., Duan Q., Wang Z., et al. Enrichr: a comprehensive gene set enrichment analysis web server 2016 update. Nucleic acids research. 2016; 44(W1), W90–W97. doi: 10.1093/nar/gkw377 27141961 PMC4987924

[45] Mordas A., & Tokatlidis K. The MIA pathway: a key regulator of mitochondrial oxidative protein folding and biogenesis. Accounts of chemical research. 2015; 48(8), 2191–2199. doi: 10.1021/acs.accounts.5b00150 26214018 PMC4551283

[46] Esnault C., Lee M., Ham C., & Levin H. L. Transposable element insertions in fission yeast drive adaptation to environmental stress. Genome Research. 2019; 29(1), 85–95. doi: 10.1101/gr.239699.118 30541785 PMC6314160

[47] Murari A., Goparaju N. S., Rhooms S. K., Hossain K. F., Liang F. G., Garcia C. J., et al. IDH2-mediated regulation of the biogenesis of the oxidative phosphorylation system. Science Advances. 2022; 8(19), eabl8716. doi: 10.1126/sciadv.abl8716 35544578 PMC9094667

[48] Freimoser F. M., Hürlimann H. C., Jakob C. A., Werner T. P., & Amrhein N. Systematic screening of polyphosphate (poly P) levels in yeast mutant cells reveals strong interdependence with primary metabolism. Genome biology. 2006; 7, 1–9. 10.1186/gb-2006-7-11-r109 PMC1794592 17107617

[49] Varela J. C., Praekelt U. M., Meacock P. A., Planta R. J., & Mager W. H. The Saccharomyces cerevisiae HSP12 gene is activated by the high-osmolarity glycerol pathway and negatively regulated by protein kinase A. Molecular and cellular biology. 1995. doi: 10.1128/MCB.15.11.6232 7565776 PMC230875

[50] Park A. Y., Park Y. S., So D., Song I. K., Choi J. E., Kim H. J., et al. Activity-regulated cytoskeleton-associated protein (Arc/Arg3. 1) is transiently expressed after heat shock stress and suppresses heat shock factor 1. Scientific reports. 2019; 9 (1), 2592. doi: 10.1038/s41598-019-39292-1 30796345 PMC6385231

[51] Jaimes-Miranda F., & Chávez Montes R. A. The plant MBF1 protein family: a bridge between stress and transcription. Journal of experimental botany. 2020; 71(6), 1782–1791. doi: 10.1093/jxb/erz525 32037452 PMC7094072

[52] Gerards M., Cannino G., González de Cózar J. M., & Jacobs H. T. Intracellular vesicle trafficking plays an essential role in mitochondrial quality control. Molecular biology of the cell. 2018; 29(7), 809–819. doi: 10.1091/mbc.E17-10-0619 29343549 PMC5905294

[53] Öhrvik H., Nose Y., Wood L. K., Kim B. E., Gleber S. C., Ralle M., et al. Ctr2 regulates biogenesis of a cleaved form of mammalian Ctr1 metal transporter lacking the copper-and cisplatin-binding ecto-domain. Proceedings of the National Academy of Sciences. 2013; 110(46), E4279–E4288. doi: 10.1073/pnas.1311749110 24167251 PMC3831961

[54] Ross J. A., Nagy Z. S., & Kirken R. A. The PHB1/2 phosphocomplex is required for mitochondrial homeostasis and survival of human T cells. Journal of biological chemistry. 2008; 283(8), 4699–4713. doi: 10.1074/jbc.M708232200 18086671

[55] Yeo J. H., Skinner J. P., Bird M. J., Formosa L. E., Zhang J. G., Kluck, et al. A role for the mitochondrial protein Mrpl44 in maintaining OXPHOS capacity. PloS one. 2015; 10(7), e0134326. doi: 10.1371/journal.pone.0134326 26221731 PMC4519308

[56] Boldogh I. R., Yang H. C., & Pon L. A. Mitochondrial inheritance in budding yeast. Traffic. 2001; 2(6), 368–374. doi: 10.1034/j.1600-0854.2001.002006368.x 11389764

[57] Xu X., & Friedman J. S. A Role for the Transcriptional Coactivator PRC1 in Mitochondrial Biogenesis During Erythroid Development. 2009

[58] Yin W., Kissinger J. C., Moreno A., Galinski M. R., & Styczynski M. P. From genome-scale data to models of infectious disease: a Bayesian network-based strategy to drive model development. Mathematical biosciences. 2015; 270, 156–168. doi: 10.1016/j.mbs.2015.06.006 26093035 PMC4679518

[59] Thorne T. Netdiff–bayesian model selection for differential gene regulatory network inference. Scientific Reports. 2016; 6(1), 39224. doi: 10.1038/srep39224 27982083 PMC5159802

